# Spontaneous Emulsification as a Function of Material Exchange

**DOI:** 10.1038/s41598-017-05861-5

**Published:** 2017-07-14

**Authors:** Stephen Spooner, Zushu Li, Seetharaman Sridhar

**Affiliations:** 0000 0000 8809 1613 grid.7372.1 WMG, University of Warwick, Coventry, CV4 7AL UK

## Abstract

Direct visualization at 1873 K of 0% to 8% molten FeAl droplets suspended in a SiO_2_ enriched oxide medium was carried out to image the evolution of droplet morphology during reaction between Al and SiO_2_. Phenomena such as perturbation growth, necking and budding of offspring droplets from a bulk body are observed. The observations are used to discuss and inform a new approach to the nature of interfacial tension and the impact this has on concepts used to define interfacial tension for a two phase system with material exchange across the interface. The mapping of global interfacial tension coupled with free energy dissipation has been used to give an energetic reasoning as to the behaviour seen with respect to aluminium content in the metal phase.

## Introduction

Interfacial tension between molten oxide slurries and liquid steel controls the rate of reaction where material exchange between the two phases is concerned. The steps involved in the transfer of material between the two phases are:

1. Mass transfer of a reactive oxide species to the interface in the molten oxide phase
2. Mass transfer of the reactive element to the interface in the liquid metal phase
3. Interaction of the respective species at the interface
4. Mass transfer of the reaction product away from the interface in the respective phase

The specific system interrogated within the present work is of particular pertinence in manufacturing due to the social drive of lightweighting in sectors such as the automobile industry. As a result the steel industry finds itself in a position where the development of high aluminium content grades such as TRIP and low density products is no longer a luxury but a necessity in order to stay competitive in the market in comparison to materials such as light metals, composites or carbon fibre.

A high aluminium alloyed steel offers the potential for a stronger lighter material in comparison to those currently available. However, such an alloy presents product issues such as grain refinement due to the lack of phase change, as well as processing chemical stability with both ladle slurries and mould flux compositions due to the high SiO_2_ content favourable for both viscosity and melting point influences. Processing flow and the addition of material are known to cause droplet formation of the liquid metal suspended through a protective or partitioning oxide phase, resulting in a large increase in reactive surface area and a potential for significant material exchange.

In the case of aluminium transfer from liquid steel to a metal oxide phase the species involved are elemental aluminium and silicon dioxide; the reaction is shown in equation 1:

1 $$4[{\rm{Al}}]+3({{\rm{SiO}}}_{2})\to 3[{\rm{Si}}]+2({{\rm{Al}}}_{2}{{\rm{O}}}_{3})$$

where species in [] are in within the liquid metal phase, and those in () are in the molten oxide phase. In systems such as these at liquid temperatures it is well documented that the reactions at the interface are much faster than material mass transfer within the bulk phases. As such, a system of two liquid phases undergoing reaction towards equilibrium may facilitate an increase in the speed of the reaction through reduction of the diffusion length from bulk to interfacial reaction site. This has previously been reported as a broadening of a metal droplet during sessile drop interrogation^1^ (reduced contact angle) or in the case of an unbound metal droplet mixing of the two phases into a micro emulsion^2, 3^.

With these conditions the reaction rates of a system where a defined first phase (in this study the metal droplet) is freely suspended in a continuous second phase (in this study the metal oxide mixture) are limited by the interfacial area/bulk volume ratio, with the size of the interface (or surface area of a single material) being a balance between material size, interfacial tension and physical forces.

## Theoretical Consideration of Interfacial Tension

Interfacial tension (γ) is a balance of the favourable homogeneous phase interactions as opposed to heterogeneous phase interactions; this is shown through equation 2:

2 $$\gamma =\,(\sum a{E}_{i}^{1-1},b{E}_{i+1}^{1-1},c{E}_{i+2}^{1-1}\ldots x{E}_{i+n}^{1-1})-(\sum d{E}_{i}^{1-2},e{E}_{i+1}^{1-2},f{E}_{i+2}^{1-2}\ldots y{E}_{i+n}^{1-2})$$

where ${E}_{i}^{1-1}$ is the homogeneous phase interaction within the i^th^ solvation shell; ${E}_{i}^{1-2}$ is the heterogeneous interaction within the i^th^ solvation shell; and a, b, c, d … x, y are scaling factors for the number of interactions experienced with each solvation shell. Within a defined interface a > b and so on for the scale of each solvation shell. It should be noted from the relationship described in equation (2) that interfacial tension can never be zero. Homolytic and heterolytic interaction will never be equal in energy. If heterolytic interaction is stronger than homolytic; equation 3 would become true:

3 $$\sum _{i}^{n}{E}^{1-2} > \sum _{i}^{n}{E}^{1-1}$$

if true there is favourable free mixing of species, there is no defined interface, only a single phase. From this we see interfacial tension minimization is driven by an increase in free energy as atomic interaction energies are fixed for a given condition. This results in the driving force of surfactant behaviour arising if possible where heterogeneous interaction between the interfacial location driven species and a bulk phase is less of an energy cost. For iron and metal oxide two phase systems, known atomic interfacial active species of significance are O, S and P in descending order of influence^4, 5^. The concentration of these species with respect to interfacial energy reduction has been extensively studied; the present authors have shown the driving of this species to an equilibrium state throughout an industrially relavant system being a key contributing factor to the spontaneous emulsification of a metal droplet^2, 3^.

As well as the doping of an interface with surfactants the authors will investigate if material exchange across the interface alone can be enough to reduce “effective” interfacial tension to a point where physical forces such as Marangoni flow can be enough to cause the break-up of an interface.

**Figure 1:**
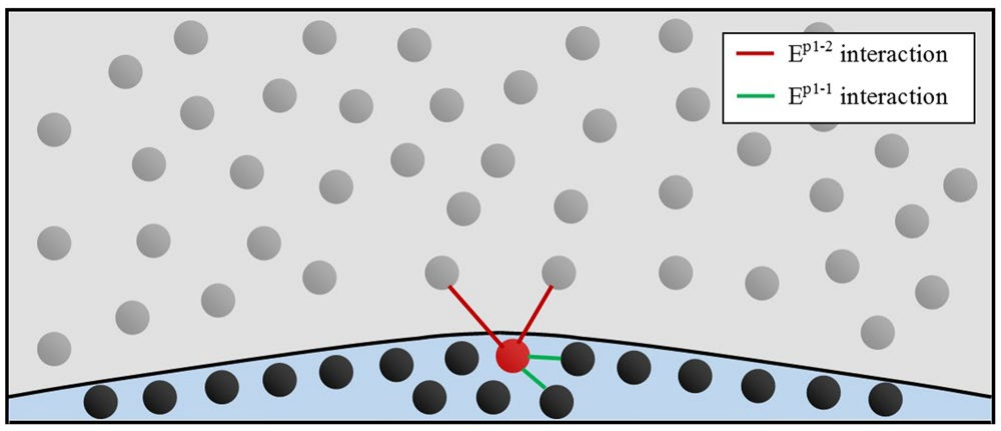
A Schematic of the interface between a molten iron droplet (black particles) and a molten oxide suspension medium (grey particles). An Al atom is present at the interface (red) with examples of inter (E^P1-1^) and intra (E^P1-2^) phase interactions depicted.

Movement of species across an interface due to thermodynamically favourable reaction is a manifestation of the ${E}_{i+n}^{1-2}$ term increasing from equation 3. In the case presented here, transfer of aluminium across the interface can be seen as a sublattice of aluminium kinetically partitioned from the thermodynamically stable oxide phase, where the iron-aluminium interactions dictate the rate of aluminium movement through the bulk metal phase and across the interface (the diffusion rate). An example of when equation 4 holds true is if phase homolysis is considered as opposed to atomic homolysis:

4 $$\sum _{i}^{n}{E}^{P1-2}+f(\Delta {S}_{mix}) > \sum _{i}^{n}{E}^{P1-1}$$

where E^P1−2^, E^P1−1^ are representative of the interaction energies of aluminium with the bulk oxide phase and bulk metal phase respectively depicted in Fig. 1. In addition a function of the entropy of mixing (f(∆Smix)) should be considered as shown within equation 4. When an aluminium atom reaches the interface, it must displace/fill a void of the bulk phase locations (in this case iron atoms), reducing order at the interface and the number of E^1-1^ interactions an iron atom experiences at the interface; an impurity at the interface reduces interfacial tension^6^.

In this paper we will interrogate the starting concentration of aluminium atoms in the metal drop (which governs the flux of aluminium atoms to the interface) required to cause sustained perturbation growth on the metal phase surface, as well as the number required to facilitate spontaneous emulsification of the metal phase through a molten oxide matrix. The specific system of aluminium reacting with silica in the oxide phase is not only chosen due to its direct relevance to current technology advancements, but also has the benefit of a transparent oxide phase being sustained throughout the experiment due to lack of transition metal oxides being formed (as would be the case if for example the reaction with manganese was studied; as manganese oxide entered the slag phase its opacity would slowly increase, blocking observation of the droplet later in the reaction), and thus allows for the continuous observation of the metal droplet phase as it transpires through the full spontaneous emulsification life-cycle.

## Materials and Methods

In this investigation seven FeAl alloys were used; their compositions are presented in Table 1. The alloys were formed using an open induction furnace with a stock electrically produced “pure” iron chip source and the addition of high-purity aluminium chips with consecutive addition and sampling of the melt to screen the required compositions. All experiments used the same master oxide mixture, the composition of which is presented in Table 2. The oxide phase was produced from powder mixing of the three reagent grade powders followed by pre-melting of the mixture at 1873 K in a horizontal tube furnace using a gaseous environment of argon (<2 ppm oxygen). The pre-melting stage was used to ensure full mixing of the oxide compounds, and was followed by disc milling to form a well-mixed master oxide phase.

**Table 1:** The compositions of FeAl alloys used for the experiments in HT-CSLM presented in mass %. Compositions were measured by ICP.

Sample ID	Mn%	P%	Ni%	Cr%	Al%	C%	S%	O%	N%
0% Al	0.0003	0.0004	0.0001	0.0003	0.00005	0.004	0.001	0.0034	<0.001
1% Al	0.0003	0.0004	0.002	0.0005	0.99	0.0011	0.001	0.0028	<0.001
2% Al	0.0004	0.0004	0.001	0.0003	1.92	0.0006	0.001	0.0029	<0.001
3% Al	0.0002	0.0004	0.002	0.0005	2.84	0.0011	0.002	0.0035	<0.001
4% Al	0.0004	0.0004	0.002	0.0005	3.98	0.0007	0.002	0.0042	<0.001
5% Al	0.0003	0.0003	0.002	0.0004	4.92	0.0009	0.002	0.0040	<0.001
8% Al	0.0002	0.0004	0.002	0.0005	7.87	0.0007	0.001	0.0038	<0.001

**Table 2:** The composition of the metal oxide mixture used for HT-CSLM experimentation after pre-melting of reagent grade hand mixed powders presented in mass %. Compositions were measured by XRF.

CaO%	SiO_2_%	Al_2_O_3_%
36.17	23.11	38.53

**Figure 2:**
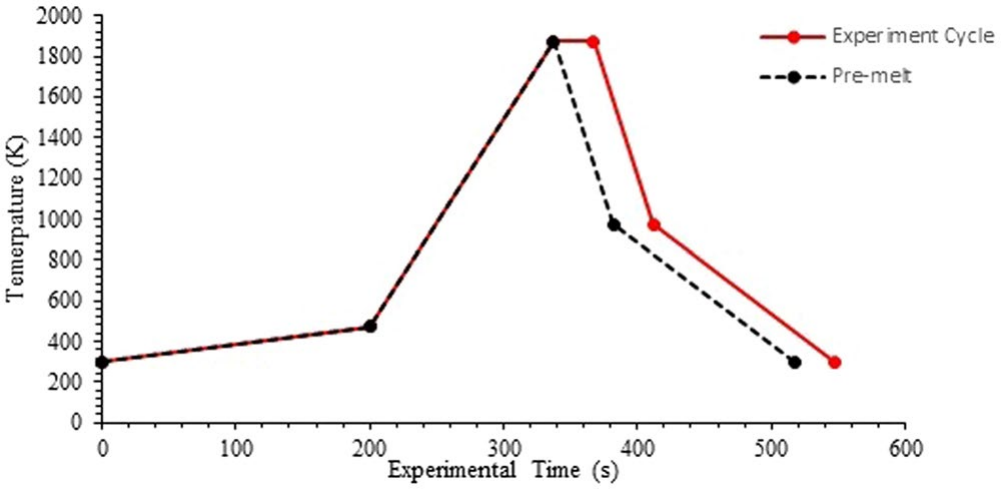
The nominal heating cycle used for both pre-melting of metal oxides and experimentation of oxide/metal alloy reaction in the HT-CSLM.

A high-temperature confocal scanning laser microscope (HT-CSLM) was used to perform the *in-situ* observation of the reacting phases. Details on the microscope can be found in previous reportings^3^. 0.2 g ± 0.02 of the oxide powder was placed into a sapphire crucible. After placing of the sample inside the HT-CSLM chamber, the chamber is evacuated using a rotary pump for half an hour, followed by back filling with high-purity argon (99.9999% argon passed through a further heated getter containing copper and magnesium chips at 623 K); this is done three times to ensure a clean environment with a minimal oxygen partial pressure in the chamber. This initial sample is then pre-melted at 1873 K in the HT-CSLM following the regime depicted in Fig. 2. Upon cooling to room temperature the oxide forms a glassy phase with a deep meniscus due to high wettability of the liquid oxide with the crucible walls. A cylinder of mass 17 mg ± 0.7 (a large mass range is due to the varying density of the alloys) with dimensions 1.19mm H, 1.49mm D (professionally machined) of the respective alloy is then added to the centre of the glass meniscus and a further 0.3 g ± 0.12 of oxide powder is placed on top of the metal and lightly compressed by hand. The crucible is then placed back into the HT-CSLM on top of a platinum foil spacer which in turn is on top of an alumina spacer. The platinum spacer is used to increase back scattering of light through the optically transparent molten oxide phase at experimental temperature (increased field of view in the z-direction); the alumina spacer is used to stop the platinum spacer sticking to the platinum ring of the sample stage. This chamber is again cycled three times through the vacuuming and argon back filling stages before the experiment is conducted under the melting profile displayed in Fig. 2. Due to the dynamic environment produced within the HT-CSLM sample it is very challenging to locate the metal droplet; high levels of light gain (widening of the UV laser aperture) and brightness are used to locate the droplet between the slag melting point and the beginning of meaningful video recording before settings are returned to those suitable for imaging. Video was captured at 15 fps.

Calibration of the HT-CSLM is conducted through the melting of silver, copper and iron samples and aligning the temperature readout with the actual melting temperature of these samples. Under such setup the thermocouple of the HT-CSLM can be as much as 70 K away from the true sample temperature when investigated in this manner. Figure 2 and reported experimental temperatures are after calibration.

With the use of the semi-transparent sapphire crucibles the physical heating profile of the HT-CSLM was also investigated. Pieces of thermal paper (similar to that used in till receipts) cut to size were placed in the base of the crucible on the stage and spacers. The halogen bulb was then manually turned on for short periods of time and the resulting heat markings on the paper observed. At low bulb powers and very short times a 2 mm diameter spot in the centre of the crucible was observed, this represents the designed focal point of the HT-CSLM chamber. With incremental increases in time and power exposure, the centre point in the paper was not seen to grow by an amount observable by eye (due to the poor thermal conduction of the paper), however a ring around the outside of the crucible was seen to form and grow inwards. In comparison an opaque crucible such as one of the same dimensions formed of high-density MgO, did not display the outer heat ring on the crucible base. It is thought that the transparency of the sapphire crucibles to the IR wave lengths emitted by the halogen bulb allows for direct heating of a sample not only from the centre focal point but also from the crucible edges inwards. This creates a more uniform heat profile through the sample than in previous experiments, reducing convection and aiding the metal droplet to stay relatively stationary for viewing during reaction.

The videos produced from HT-CSLM experiments have undergone image processing through grey scale correction to a two-point grey scale, followed by a segmentation of the image through manual determination of white dictating in-focus metal phase, and black dictating molten oxide phase. In the case of observations such as a large black section/hollow surrounded by the light area of the droplet, this has been included in the segmented area representing the droplet. Data produced from this method was averaged over 3 frames (the frame each side and the frame of the time point reported) due to the raster of confocal focus point in the z-plane to give clearer imaging and greater accuracy in determination of surface area calculation.

**Figure 3:**
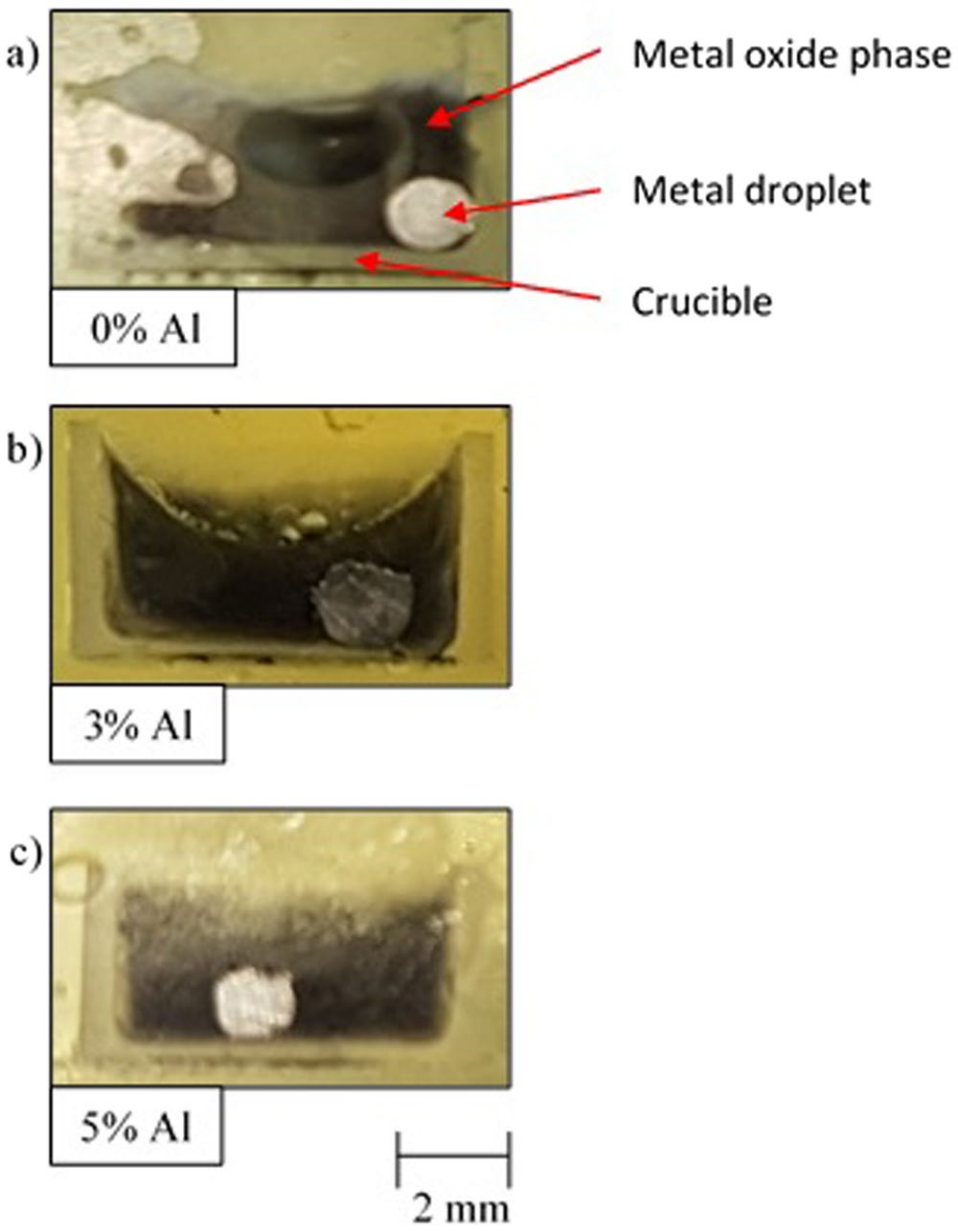
Images of the sectioned quenched samples showing the droplet suspended in the oxide phase. As well as the reduction in metal recovery in higher Al content systems. (**a**) 0% FeAl, (**b**) 3% FeAl, (**c**) 5% FeAl.

Further to this, samples were mounted in resin and sectioned to observe the quenched material. Figure 3 shows the cross sections observed by eye of quenched samples originally containing the 0%, 3% and 5% FeAl alloys. From these images we can clearly see that the droplet is suspended in the oxide phase (not on the surface). In addition it is qualitatively notable that there is a reduced mass of material recovered when the initial FeAl content has been increased (a-0%, b-3% and c-5%). This is expected due to the higher droplet surface area produced during the experiment allowing for a larger quantity of Fe to be oxidized (causing the metal phase volume reduction), as well as the additional factor of offspring droplets having not fully coalesced in the emulsifying systems.

## Results

**Figure 4:**
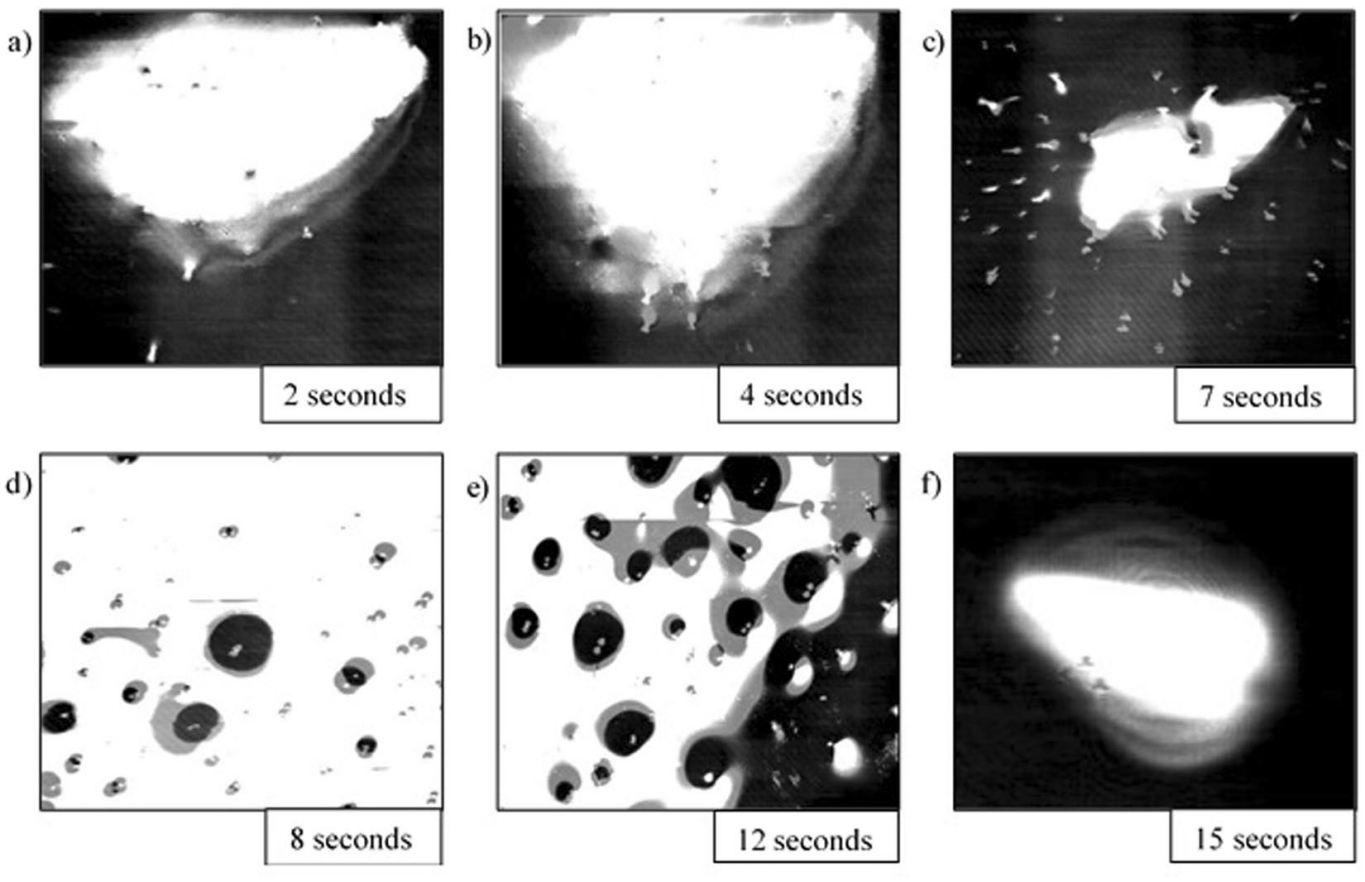
Time step images of the 5% FeAl experiment as seen in the HT-CSLM. In focus metal phases appear as lighter in grey scale and the molten metal oxide phase is seen as black. (**a–f**) Shows the cycle of perturbation-emulsification and finally coalescence.

**Figure 5:**
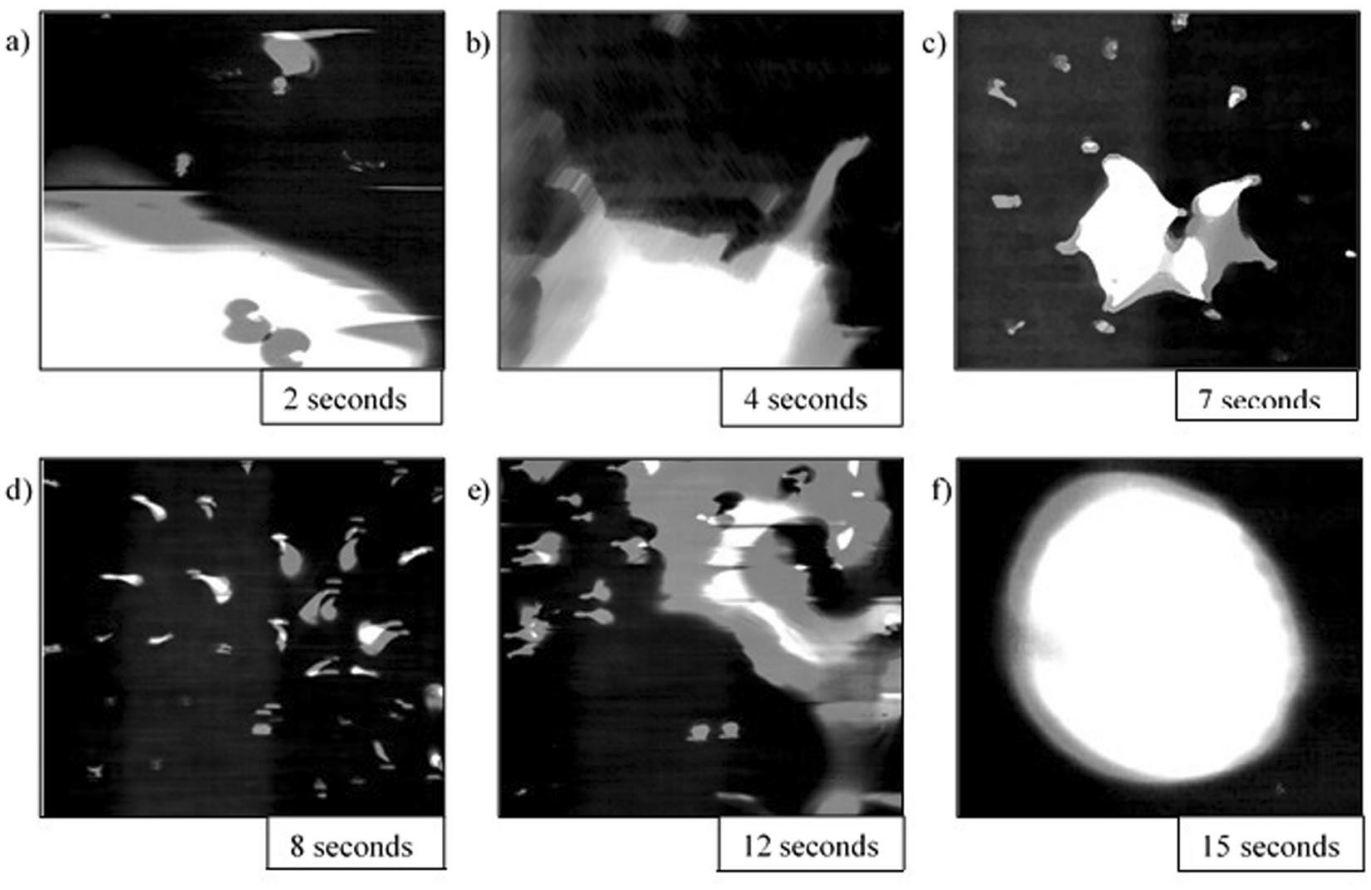
Time step images of the 4% FeAl experiment as seen in the HT-CSLM. In focus metal phases appear as lighter in grey scale and the molten metal oxide phase is seen as black. (**a–f**) Shows the cycle of perturbation-emulsification and finally coalescence.

**Figure 6:**
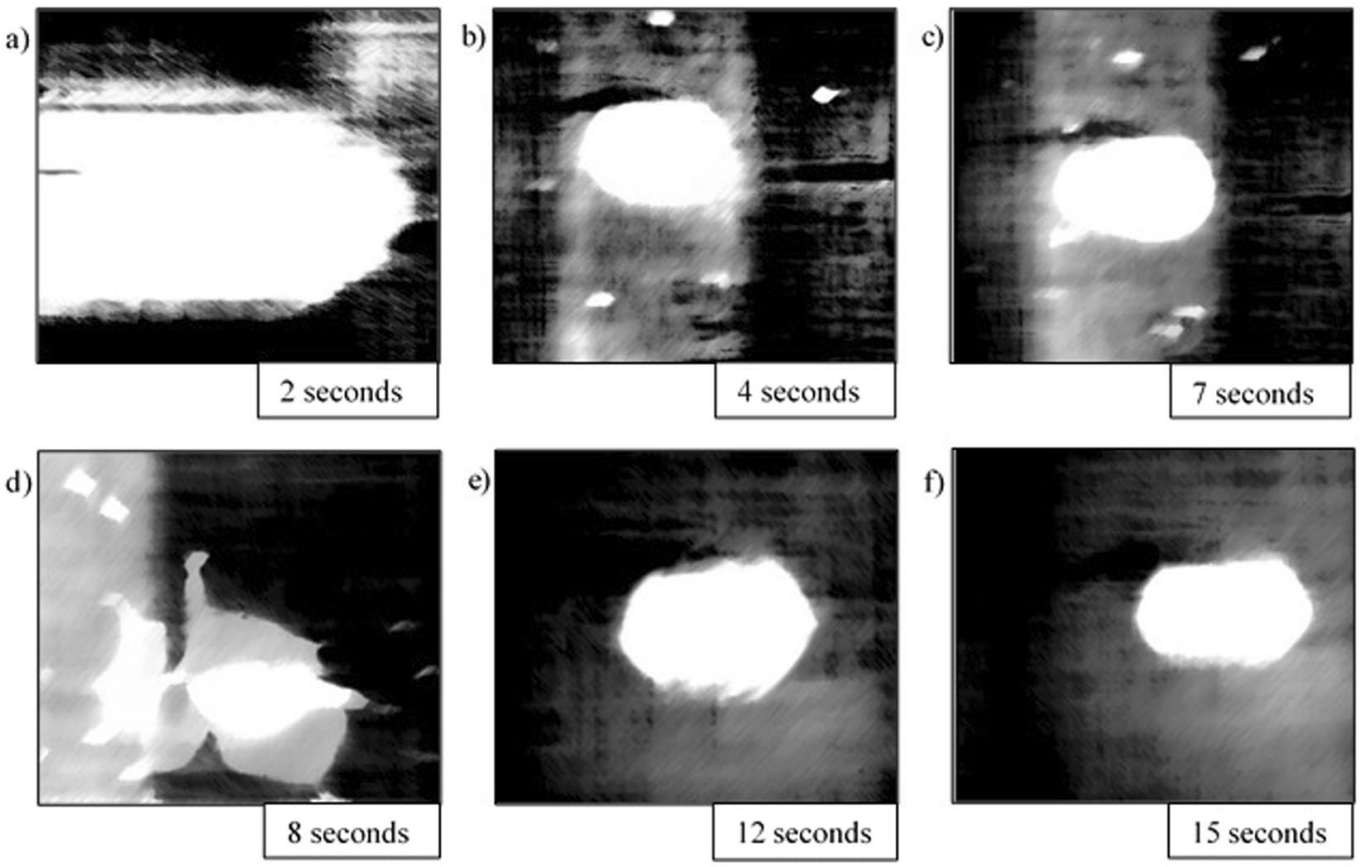
Time step images of the 3% FeAl experiment as seen in the HT-CSLM. In focus metal phases appear as lighter in grey scale and the molten metal oxide phase is seen as black. (**a–f**) Shows the cycle of perturbation growth with no emulsification seen.

**Figure 7:**
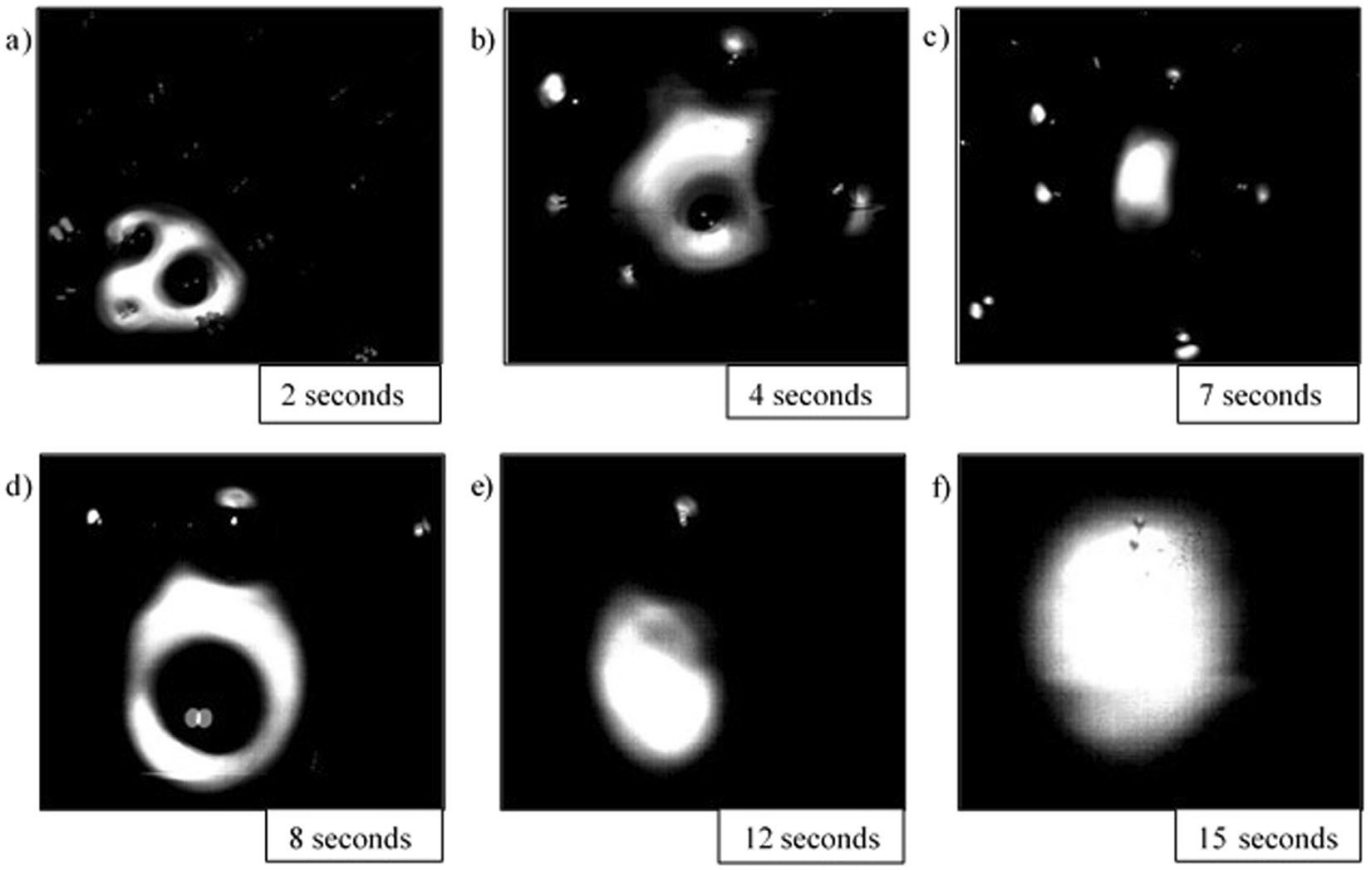
Time step images of the 2% FeAl experiment as seen in the HT-CSLM. In focus metal phases appear as lighter in grey scale and the molten metal oxide phase is seen as black. (**a–f**) Shows the cycle of perturbation growth to quiescent droplet.

**Figure 8:**
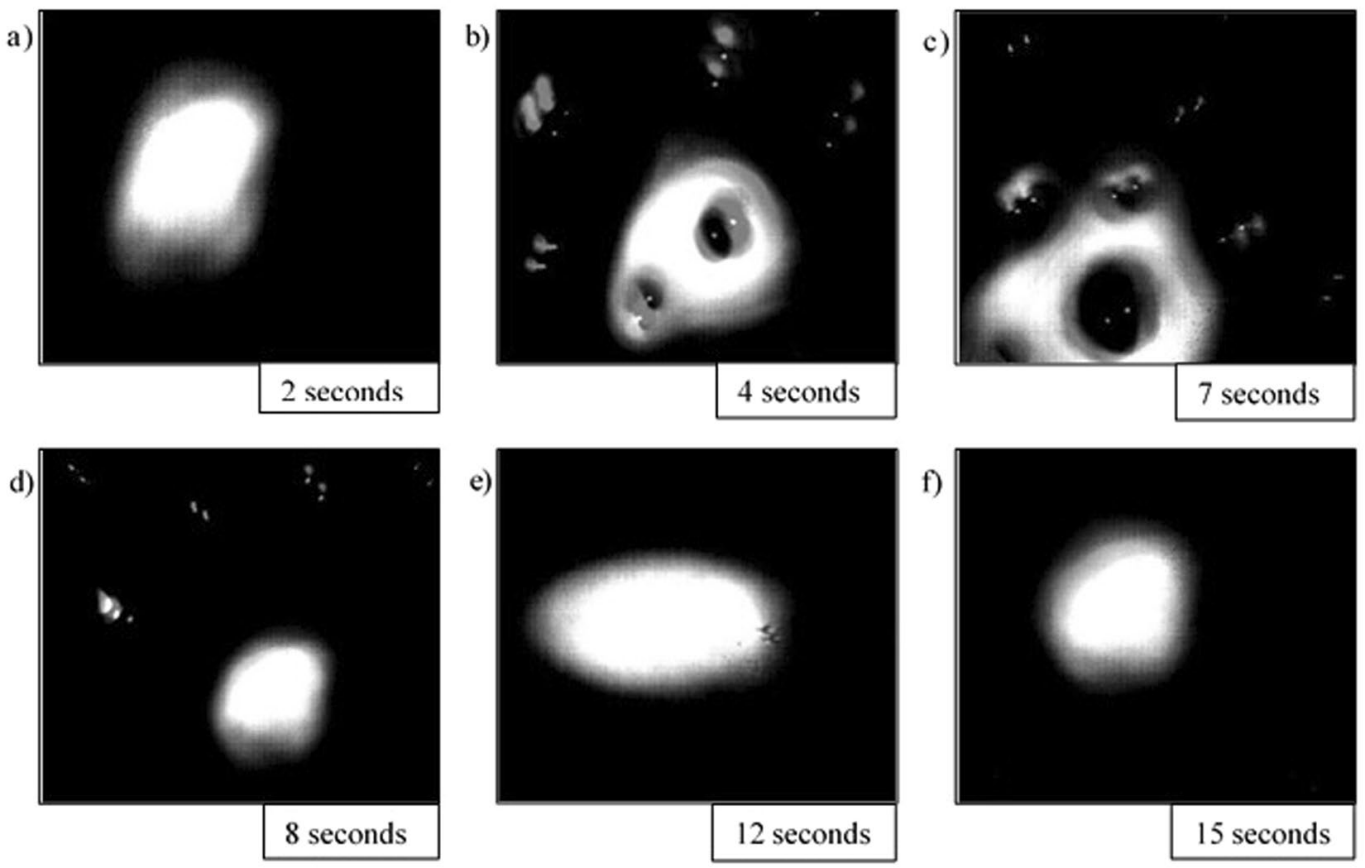
Time step images of the 1% FeAl experiment as seen in the HT-CSLM. In focus metal phases appear as lighter in grey scale and the molten metal oxide phase is seen as black. (**a–f**) Shows the cycle of perturbation growth to quiescent droplet.

Stills of the video output from the HT-CSLM are presented in Figs 4, 5, 6, 7 and 8. These figures correspond to samples 5% FeAl through to 1% FeAl in respective descending order. Images from 8% FeAl show a very similar behaviour to that of 5% FeAl, and those of 0% FeAl show a quiescent near-spherical droplet for the entire period.

Figure 4 shows the time-dependent behaviour of the 5% FeAl sample. From the point at which the droplet is focused upon, perturbation levels are seen to exponentially increase to a point where the entire field of view is flecked with points at which perturbations from lower down in the sample have grown into the focal level. At 8 seconds the metallic phase clouds the entire field of view as seen in Fig. 4d (due to the 2D imaging of the 3D system individual droplets of the formed micro emulsion are indistinguishable). The system then goes on to begin coalescence of the metal droplets at 12 seconds where towards the edge of the sample defined small pockets of isolated metal phase can be seen, before complete coalescence is observed at 15 seconds after temperature is reached.

Figure 5 is the time-step image sequence from the 4% FeAl sample with the same image times as that of Fig. 4. It is quantitatively observable that the level of perturbation observed in Fig. 5a,b and c is lower (there are a lower number of distinct perturbations) than that of the 5% FeAl sample with the sample emulsification seen to be delayed; emulsification is only visible in the 12-second still and the level of emulsification is seen to be less than that of the 5% FeAl sample.

In Fig. 6 the sample time-steps are presented for the 3% FeAl sample; in this video no clouding/emulsifying behaviour was observed. The level of perturbation is much reduced compared to that of the images preceding emulsification in the 5% and 4% FeAl samples, however the period of highest perturbation levels is still in the region of 8 seconds. The sample is however seen to be quiescent at 12 seconds.

The cycle of the 2% FeAl and 1% FeAl samples during reaction can be seen in Figs 7 and 8 respectively. The images show significantly reduced levels of perturbation compared to that of the 3% FeAl sample, and a shift of the highest level of perturbation to earlier in the experimental regime (around 7 seconds). The difference in behaviour is difficult to qualitatively justify from viewing of the video, however after image processing and subsequent measurements being performed there is a subtle but definite reduction in perturbation level and sustained perturbation time between the 2% and 1% FeAl samples.

**Figure 9:**
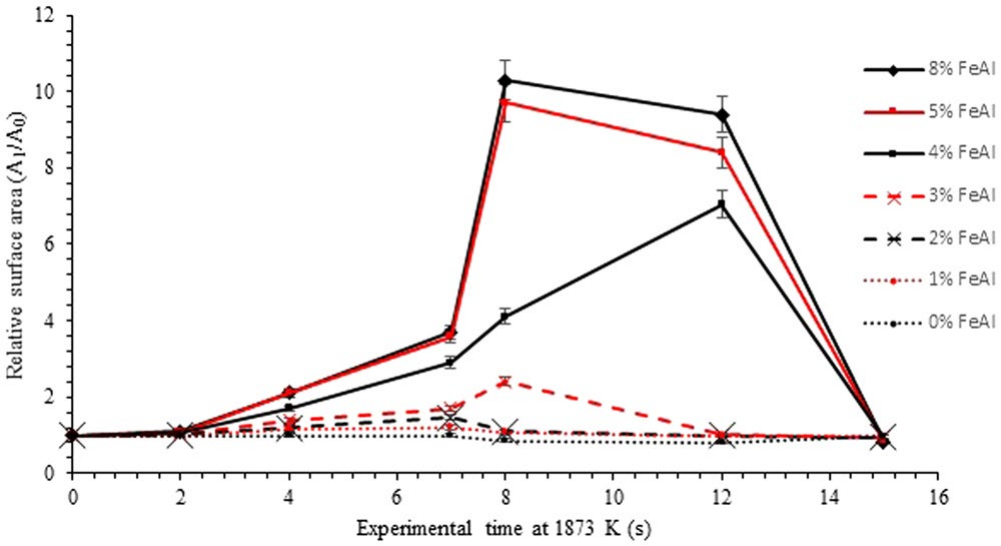
The surface area ratio profiles as deduced from image analyses of the HT-CSLM video (to the nearest 0.05). 1 in 15 frames have been analysed but only the points corresponding to those in Figs 3, 4, 5, 6 and 7 are highlighted.

Image processing allows for quantification of the liquid metal surface area as seen in the 2D images of the HT-CSLM. Figure 9 displays the relative metal surface area change as a function of time for all seven experiments (extrapolated back to zero time assuming a quiescent sphere is formed on first melting). The measurements of the images displayed in Figs 4, 5, 6, 7 and 8 are highlighted.

From Fig. 9 we can see that the 2D image analysis has led to distinct profiles for different levels of Al in the metal droplets. Both 8% and 5% FeAl samples show an emulsification profile near 10 times the original surface area of an ideal droplet (spherical shape) at its highest point; they both peak at 8 seconds. The 4% FeAl sample still shows emulsification behaviour to a lesser extent with a maximum of near 7 times an ideal droplet; this maximum does however come significantly later in the reaction process. Samples of 3%, 2% and 1% FeAl show much lower levels of surface area increase, with less perturbation than the higher aluminium content alloys and no emulsification behaviour. Although not visible due to scale on the graph here, there is a trend of end-point surface area ratio being below 1, with samples that emulsified having a lower ratio than those that didn’t. This reduction in size is due to the oxidation of the sample/dissolution into the slag phase. Samples which emulsified have much larger surface area not only for aluminium refinement but also for oxidation of the bulk iron phase, meaning greater loss of material (as previously depicted in Fig. 3).

## Discussion

Figures 4, 5, 6, 7 and 8 clearly depict the change in droplet morphology lifecycle when aluminium content is reduced in the metal droplet. The quantification of surface area change shown in Fig. 9 offers clarification of four behaviours. The 8% and 5% FeAl samples show exponential increase in surface area between 7 and 8 seconds. This large increase in area over such a short period shows true spontaneous emulsification behaviour; the droplets effectively tear themselves apart into the micro-emulsion of smaller droplets. This may be indicative of breakaway droplets further breaking down due to the large chemical potential in place across the interface. The 4% FeAl sample shows a similar level of emulsification to the higher aluminium content samples, however the peak in surface area is later into the reaction cycle and the increase is more gradual. A method by which this would happen is if the budding of perturbations on the droplet surface continued to occur at a faster rate than any coalescence; rather than a sudden break down of the droplet there is a gradual release of offspring droplets through the reaction period.

Although the 8%, 5% and 4% FeAl samples show differing emulsification profiles, the coalescence behaviour is very similar. Due to the differing time scales for emulsification to occur, the severity of emulsification and the sustained chemical potential across the interface in the offspring droplets aluminium content is able to reduce at a faster rate in the metal droplet at higher original aluminium content. So much so that we see all emulsifying systems coming to a quiescent droplet within a very close time proximity.

Riboud and Lucas conducted similar experiments where a larger iron-aluminium alloy droplet was observed via x-ray radiography. There results were recorded over a much longer time period (around 50 minutes), and their interpreted interfacial tension between metal and oxide phase during the observation is given. They follow the workings of Defay, Sanfeld, Steinchen and Friedel^7–9^, where a model of equidistant planes parallel to the interface containing the molecules is considered against a perpendicular z diffusion axis. In this model they discuss a mechanical equilibrium expression for interfacial tension such that:

5 $$\gamma ={e}^{3}{\int }_{a}^{b}\sum _{x}\sum _{x^{\prime} }{k}_{x{x}^{^{\prime} }}{c}_{x}^{^{\prime} }{c}_{{x}^{^{\prime} }}^{^{\prime} }dz$$

where ${k}_{x{x}^{^{\prime} }}$ is the attraction between molecules x and x’ situated in a layer thickness e; ${c}_{x}^{^{\prime} }=d{c}_{y}/dz$ and ${c}_{{x}^{^{\prime} }}^{^{\prime} }=d{c}_{{y}^{^{\prime} }}/dz$ are the concentration gradients along the z-axis; a and b are positions on the z-axis in two phases A and B far enough from the interface that c’_y_, c’_y’_ are negligible. The result of this is discussion of interfacial tension being zero when there exists a pair of concentration gradients with opposite signs and heterogeneous interactions are greater in strength than homogeneous interactions. Although this is similar to the treatment of interfacial tension discussed in the opening arguments, there is a fundamental difference where the current work considers this movement of favourable exchange between phases to be a sublattice exchange; this is not the mixing of the two bulk phases. If two bulk phases still exist, there must be a net positive interfacial tension.

This being said, the trend of interfacial tension change does not change, only the absolute values. These values are incomputable from the data collected in this study, however if the trend reported by Riboud and Lucas^10^ is corrected for time scale due to the miniaturization the present study the interfacial tension coincides well with the interfacial area changes in current systems, with the spontaneity of emulsification and coalescence captured. There is a delay of surface tension increase in comparison to that of interfacial tension in the earlier study which is thought to be caused by the heating performance of the HT-CSLM. During these early stages the sample in the HT-CSLM may be below the programmed temperature of 1873 K and spatially heterogeneous with regards to temperature, as such reaction between aluminium and SiO_2_ is hindered.

Both Riboud *et al*.^10^ and Rhamdhani *et al*.^11–13^, present the most directly relevant system reportings in the literature previously. The major experimental differences between the three studies are: Riboud^10^ uses a larger droplet dimension (0.5–2 g) and the experiment is akin to *in-situ* x-ray observation of a sessile experiment, the droplet is in contact with a substrate at all times; Rhamdhani^11^ uses a larger droplet dimension (1.7–3.25 g), with the droplet suspended in the oxide medium and samples were quenched and crushed for evaluation; the present study uses smaller droplets (17 mg ± 0.7), suspended in the oxide medium observed *in-situ* via a scanning UV laser.

**Figure 10:**
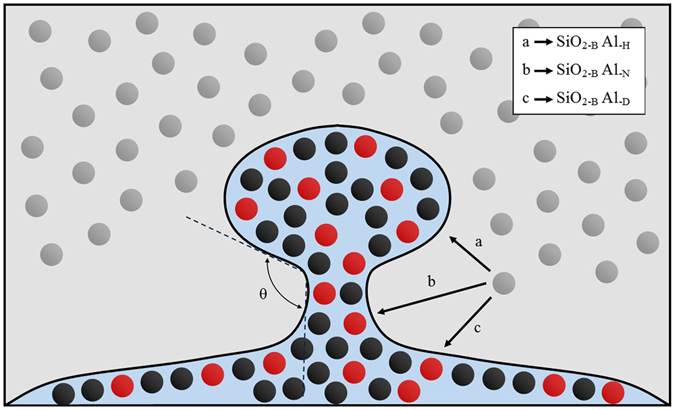
A schematic of the interface between an iron aluminium metal alloy (black particles represent iron atoms, red particles represent aluminium atoms) and a metal oxide medium (specifically silica molecules, grey particles). An example of perturbation growth and necking is given with varying diffusion distances of SiO_2_ bulk particles to perturbation head (SiO_2-B_ Al_-H_, neck (SiO_2-B_ Al_-N_) and bulk droplet (SiO_2-B_ Al_-D_) are qualitatively depicted. The strain angle as a result of necking is also indicated.

The first consideration for analysing the effects of experimental differences is the pathway of perturbation growth and budding of the system. Perturbations initially grow due to the shortening of diffusion distance to reactive species in the bulk oxide phase. Figure 10 offers a schematic tool to explain this, where: a) depicts the diffusion distance for SiO_2_ to reach the perturbation head from the bulk oxide phase; b) depicts the diffusion distance for SiO_2_ to reach the perturbation length centre (and at a later stage of growth, the perturbation neck); c) depicts diffusion distance for SiO_2_ to reach the main droplet body. As material is reacted the perturbation growth is sustained through entering areas of the bulk oxide phase where SiO_2_ is still enriched; this microscopic growth phenomenon reduces the diffusion length required for new SiO_2_ molecules to reach the interface. The sustained growth/increase in surface area is energetically rationalised by the increase in material exchange between the two phases; the Gibbs free energy is still reducing in the overall system. As the perturbation grows distance a) is maintained, and distance c) is maintained, but the effective distance b) (distance from the perturbation centre to the bulk oxide phase) is increased due to depressed level of SiO_2_ in the oxide bulk phase through which the perturbation front has previously grown. This creates a profile of reaction potential down the perturbation, with higher chemical potential in the perturbation head and lower chemical potential further down the direction of growth. This results locally in a necking phenomenon as the advancing perturbation head growth is sustained while there is much less drive for increased width close to the main droplet body. This results in the development and gradually increasing severity of the angle labelled θ on Fig. 10. We can add this geometric constraint to the rudimental expression for interfacial tension presented earlier in this work together with mixing drive in the system to give an expression for inter-phase tension γ_p_:

6 $${\gamma }_{p}=g(\theta )(\sum a{E}_{i}^{1-1},b{E}_{i+1}^{1-1},c{E}_{i+2}^{1-1}\ldots x{E}_{i+n}^{1-1})-(\sum d{E}_{i}^{1-2},e{E}_{i+1}^{1-2},f{E}_{i+2}^{1-2}\ldots y{E}_{i+n}^{1-2})-f({\rm{\Delta }}{S}_{mix})$$

where g(θ) is the geometric strain on the homolytic interactions and f(∆S_mix_) is a function of entropic mixing.

The differing results found in each experiment can thus be explained through interrogation of the curvature stress caused on the droplet while undergoing reactions. When the metal is in contact with an inert substrate as in Ribouds report^10^, the droplet shows a preference to wet the substrate (this effect was also seen in the observation of unsuccessful experiments in the present study where the droplet touched the crucible wall or base). The result of the contact is that when a perturbation grows there is little resistance from the bulk droplet (little strain) so perturbation necking is less severe, giving a reduction in geometric strain on bonding interaction at the interface. If the converse is considered (the present work), a smaller droplet in free space results in a more acute angle as θ is reduced with the perturbation neck merging with the main droplet curvature. This creates high levels of strain at the perturbation neck which are reduced and the system free energy reduction is aided by the physical detachment of the perturbation head from the main droplet body. This is the phenomenon referred to as budding.

The levels of perturbation/emulsification follow this trend through Riboud and Lucas^10^ who report a wavy quenched interface geometry for a sample in the middle of the reaction; Rhamdhani *et al*.^13^ reported some material break up through the discovery of several metal droplets of varying sizes in the quench sample; the present work shows full emulsification of the higher aluminium content samples, but also behaviour similar to those of larger droplets when lower aluminium contents are investigated (this is due to the reduction in perturbation growth speed, giving greater time for diffusion of SiO_2_ towards the perturbation length centre, reducing the impact on necking severity).

As stated earlier the time taken to reach a quiescent droplet interface at/near equilibrium is greatly reduced in the current work in comparison to that of Riboud and Lucas^10^. This is expected to be due to three reasons 1) the droplet being much smaller, 2) spontaneous emulsification occurring, greatly increasing the surface area/volume ratio and 3) an unbound droplet being able to grow perturbations into new bulk composition areas of oxide phase in all directions. These factors change the probable rate-controlling step from SiO_2_ mass transport in the earlier reported work to Al mass transport in the current findings.

The effect of oxygen concentration in the metal droplet of similar two-phase systems is well documented by several authors^4, 14–17^. Reports range from a surface tension of close to 1500 mNm^−1^ at near zero oxygen content and follow an exponential decay in interfacial tension to values of near 400 mNm^−1^. From the authors previous work we know that barriers close to the lower end of this spectrum are possible to overcome and emulsification occurs to a high degree^2, 3^.

This allows us to confirm that interfacial tension of the 3%, 2% and 1% FeAl samples did not reach levels as low as 400 mNm^−1^ as emulsification did not occur. Further to this, the current study contains no FeO in the oxide phase, a key agent in the provision of oxygen content for low interfacial tension. Although we cannot bound the interfacial tension for higher levels of aluminium, it is improbable when considering the behaviour shown with oxygen content that a 1% increase in aluminium content would drastically reduce the interfacial tension to a near zero or negative value.

In an effort to quantify the interfacial tension through visualization of spontaneous emulsification performance, the authors believe a future investigation where iron (II) oxide content is varied in the oxide phase of experiments in the HT-CSLM could be used to validate interfacial tension in in systems undergoing transport of material across the interface.

**Figure 11:**
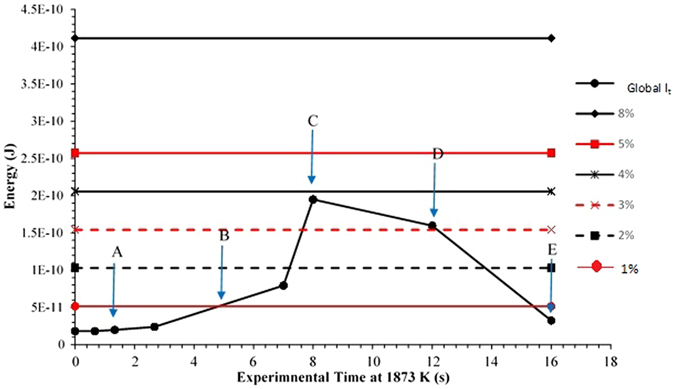
The global interfacial tension during the increase and reduction of interfacial area as measured for the 8% FeAl sample coupled with the free energy gain of reaction to equilibrium for the 8, 5, 4, 3, 2, and 1% FeAl systems. Period A is during initial light perturbation, B during heavy perturbation, C fully emulsified, D ongoing coalescence and E a fully coalesced droplet.

To quantify the reasoning behind the inflection point in emulsification behaviour of the varying FeAl content alloys, an investigation into the energy balance of free energy dissipation competing with the increased global interfacial tension due to increasing surface area is performed (values for calculations given in Table 3). Using the interfacial areas measured for 8% FeAl, (deemed the standard emulsification behaviour) and an interfacial tension of 1500 mN^m-1^ as a realistic maximum from the literature^18^, the energetic cost of interfacial area change with time can be calculated with use of equation 7 as shown in Fig. 11:

7 $$S{I}_{T}={I}_{A}\times {I}_{T}$$

where SI_T_ is the energy cost of sustaining the interface between metal and oxide in the system, I_A_ is the interfacial area between metal and oxide phases and I_T_ is the interfacial tension.

**Table 3:** The literature values used for calculation of global interfacial tension and dissipation of free energy as displayed in Fig. 11.

Interfacial Tension	1500 mN m^−1^
∆H_Formation_ Al_2_O_3_	−1669.8 kJ mol^−1^
∆H_Formation_ SiO_2_	−859.4 kJ mol^−1^
S Al_2_O_3_	50.92 J mol^−1^ K^−1^
S SiO_2_	42.0 J mol^−1^ K^−1^
C_p_ Al_2_O_3_	880 J Kg^−1^ K^−1^
Cp SiO_2_	680 J Kg^−1^ K^−1^
Temperature	1873 K

The total dissipation of free energy due to reaction for each level of aluminium content is then also calculated with use of equation 8 and displayed in Fig. 11:

8 $${\rm{\Delta }}G={\rm{\Delta }}{H}^{\ast }-T{\rm{\Delta }}S$$

where G is the Gibbs free energy, H^*^ is the reaction enthalpy calculated using Kirchhoff’s Law, T is the temperature and S is the reaction entropy.

Input values for these calculations are also presented in Table 3. At 8 seconds, the point of highest emulsification level observed in the higher aluminium content samples is pin pointed in the centre of the global I_t_ curve (zone C), with the free energy gain of the 4% FeAl sample just slightly higher. This agrees well with the experimental observations as up to zone C the surface area is growing (through regions A and B where perturbation begins, and grow), and thus a driving force for the phenomena is required. After point C we have gradual coalescence of the metal phase defined by D, restricted through statistical colocation of material and restraints such as film drain barriers (previously discussed to be dictated in a pathway well described by DVLO^3^). Thus after 8 seconds the surface area is unstable and there is no longer a driving force for a sustained interfacial area. Finally the reaction reaches point E where the droplet has fully coalesced. The 4% FeAl sample is only marginally above the energy requirements for emulsification to occur, as such this system has a minimal driving force for spontaneous emulsification which manifests as a prolonged period of time before a fully emulsified state is formed and thus the system converges towards the behaviour of the 5- and 8% FeAl samples during their respective period of slow coalesence. As the spontaneous emulfication takes longer in the 4% FeAl sample, the system will also be closer to equilibrium by the time the system reaches its highest level of emulsification; thus a slight reduction in maximum emulsification is also expected (and apparent in Fig. 9) as a higher interfacial area increase is no longer stabilized.

In addition it is possible variations within the experiment such as lagging of heating rates, temperature uniformity and packing irregularity of the slag phase have resulted in hindering the true “spontaneity” of the emulsification and the extent. This may be the cause of the droplet appearing to take longer to reach its highest emulsified state in the 4% FeAl sample, as compared to the 5- and 8% FeAl samples which are well above the required levels of free energy stabilization; overcoming any experimental hindrances.

The quantification of driving force and thus the energetic balance between reaction gain and interfacial tension cost for emulsifying phases in metal-metal oxide systems has eluded researchers in previous investigations. The direct measurement and free energy calculations discussed above, present a validated approach for the prediction of emulsification occurring when the phenomenon is dependent upon other interracially dependant reactions, such as: phosphorus and sulfur removal during steelmaking; the stability of manganese, vanadium and silicon alloying addition during ladle refining and continuous casting; and moving outside of the steel industry further examples would be iron and sulfur removal during copper smelting. Understanding of spontaneous emulsification has influence further afield in novel food production^19^, silicon particle synthesis^20^ and oil interactions with water^21^ to give a few examples.

Overall the work presents a deep understanding in the field of interfacial phenomena and the treatment of interfacial tension in a thermodynamically sound argument and presents a significant step forward in the knowledge and applicability of such phenomena in the highly competitive global steel industry and other relevant fields. The method developed allows for the *in-situ* viewing of steel and metal oxides reacting at ultra-high temperatures in 3D space and time with enough control and consistency to make accurate measurements of interfacial area change sub surface (below the surface of the metal oxide phase). This required substantial technical capability and a significant period of development.

## Conclusions

The effect of aluminium content on the transient interface morphology between iron aluminium alloy droplets and an enriched silica oxide phase has been studied *in-situ* with use of a HT-CSLM. The behaviour shows an inflection between 4% and 3% FeAl content samples evaluated through the surface area determination of the metal phase as analysed via automated post-experiment image analysis.

A discussion is given into the nature of interfacial tension where previously reported near-zero and negative interfacial tension energies are deemed unrealistic, with an alternative interpretation of the system treatment given to account for the phenomena observed.

The physical manifestation of interfacial energy change is discussed with regards to the severity of necking behaviour. This is used to evaluate the differences seen in metal morphologies between the current findings reported here and those previously reported in the literature of the most relevant systems.

Finally an interrogation of the energy balance between free energy dissipation of reaction and interfacial tension cost is given. The inflection point in behaviour relative to the aluminium content can be seen to straddle the energy cost of interfacial area increase between the 3% and 4% FeAl samples. Presenting an energetic reasoning to the observations given.
